# Occupational Monkeypox Virus Transmission to Healthcare Worker, California, USA, 2022

**DOI:** 10.3201/eid2902.221750

**Published:** 2023-02

**Authors:** Jemma Alarcón, Moon Kim, Nora Balanji, Anissa Davis, Francisca Mata, Abraar Karan, Lauren E. Finn, Annette Guerrero, McKailey Walters, Dawn Terashita, Sharon E. Balter

**Affiliations:** Centers for Disease Control and Prevention, Atlanta, Georgia, USA (J. Alarcón);; Los Angeles County Department of Public Health, Los Angeles, California, USA (J. Alarcón, M. Kim, A. Karan, L.E. Finn, A. Guererro, D. Terashita, S.E. Balter);; Long Beach Department of Health and Human Services, Long Beach, California, USA (N. Balanji, A. Davis, M. Walters);; APLA Health, Los Angeles (F. Mata)

**Keywords:** Mpox, monkeypox, monkeypox virus, occupational, healthcare worker, viruses, California, USA

## Abstract

Risk for transmission of monkeypox virus (MPXV) (clade IIb) to healthcare workers (HCWs) is low. Although many cases have been reported among HCW, only a few have been occupationally acquired. We report a case of non–needle stick MPXV transmission to an HCW in the United States.

Risk for transmission of monkeypox virus (MPXV) (clade IIb), the causative agent of mpox (formerly monkeypox), to healthcare workers (HCWs) is considered to be low, and although many cases have been reported among HCW, only a few have been occupationally acquired (*1*,*2*). We report a case of non–needle stick MPXV transmission to an HCW in California, USA.

The HCW was a 40-year-old female physician with a medical history significant for rheumatoid arthritis (treated with etanercept). In August 2022, she experienced a prodrome of myalgia and fatigue, followed by a mild headache. Two days later, she noticed a small, raised skin lesion on her left middle finger (Figure 1). The skin lesion progressed to a blister and developed umbilication; it grew to 1.5–2 cm, and a swab sample for MPXV was collected on day 6 of illness. On days 9 and 10 after symptom onset, the HCW experienced fever for 48 hours, followed by a cough and sore throat. A total of 10 skin lesions developed throughout her body, including her right arm, ankles, lateral breast, back of neck, and upper back. She received a 2-week course of oral tecovirimat, experienced no complications, and completely recovered (Figure 2).

**Figure 1:**
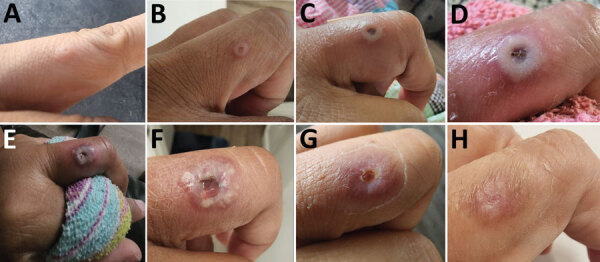
Evolution of finger skin lesions in healthcare worker occupationally exposed to monkeypox virus, California, USA, 2022. A) August 29; B) August 31; C) September 4; D) September 5; E) September 6; F) September 7; G) September 24; H) September 28.

**Figure 2:**
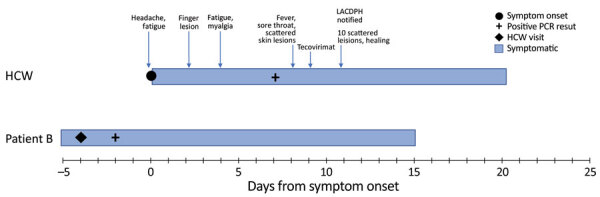
Timeline of symptom onset, testing, treatment, and public health interventions in response to a case of occupationally acquired mpox in an HCW, California, USA, 2022. Patients A and B were treated by the HCW during the presumed incubation period of her infection; however, course of illness for patient A is not shown because the HCW’s contact with patient A was >21 days before symptom onset. HCW, healthcare worker; LACDPH, Los Angeles County (California) Department of Public Health.

The Los Angeles County Department of Public Health and the Long Beach Department of Health and Human Services conducted a contact investigation. This activity was reviewed by the Centers for Disease Control and Prevention (CDC) and was conducted in accordance with applicable federal law and CDC policy.

The HCW works as a physician at 2 clinics that primarily serve LGBTQ+ and HIV-positive patients. She sees 15 patients/day and regularly sees patients with mpox, during which she wears full personal protective equipment (PPE) (N95 respirator, a gown, and eye protection). During the presumed incubation period, the HCW interacted with 2 patients (patients A and B) who did not undergo triage per clinic protocols for suspected infection with MPXV but, according to the HCW’s interview, had symptoms concerning for MPXV infection. The HCW saw patient A 29 days before her own symptom onset. She spent 15 minutes with the patient while wearing a surgical mask and gloves. When the patient disclosed symptoms concerning for mpox, the HCW left the examination room and donned PPE before swabbing the patient’s lesions. Samples from patient A tested positive for MPXV. The HCW saw patient B 4 days before her symptom onset. She spent 5 minutes with the patient while wearing a surgical mask and gloves. When the patient disclosed mpox symptoms, the HCW left the room and donned PPE before swabbing the patient’s lesions. Samples from patient B tested positive for MPXV.

Of 159 other patients seen by the HCW during the 21 days before her symptom onset, only 3 had been tested for MPXV per database cross-reference, of which 2 tested negative. The patient that tested positive had been seen by the HCW 4 days before the HCW’s symptom onset, and all lesions had healed by the visit date. Outside the workplace, investigation of the HCW’s contacts identified 1 high-risk (household) contact, 1 intermediate-risk contact, and 1 low-risk contact. High-risk and intermediate-risk contacts were offered postexposure prophylaxis. 

A site visit to the outpatient visit indicated that it has 6 examination rooms, including 1 isolation room. No sharps injuries were documented while the HCW was at clinic. The examination room is sanitized after each patient encounter. The main disinfecting products used are CaviCide disinfectant cleaner wipes and spray (Clorox, https://www.clorox.com; EPA List Q, contact time 3 min). Patients and staff share 2 gender-neutral bathrooms, which along with common areas are cleaned by building maintenance staff in the evenings. We notified 17 patients and 23 clinic staff with low-risk exposure to the HCW via calls and letters. Of the 23 staff members, 5 received postexposure prophylaxis. 

Limitations to this report include lack of acquiring phylogenetic match with contacts, no swabbing of surfaces in the clinic bathrooms or patient rooms, and no identification of secondary cases. In a previous report of occupationally acquired MPXV by HCWs in Brazil, transmission might have occurred via fomites or surface contamination during specimen collection in the patient’s home (*3*). For the HCW reported here, MPXV infection could have been acquired through inadvertent contamination during specimen collection, contact with contaminated environmental surfaces in the examination room or bathroom, or unrecognized skin contamination during glove doffing.

In Los Angeles County, HCWs who care for patients with suspected or confirmed orthopoxvirus infections, including clinicians and environmental services personnel, are now eligible for vaccination against MPXV. CDC provides guidance for specimen collection and proper PPE (*4*). In 1 study, only 23% of exposed HCWs wore all recommended PPE; but despite low adherence, no HCW had mpox develop during the 21-day incubation period (*2*). Although the risk to HCWs in the United States continues to be very low, it is crucial to continue public health outreach, infection prevention, and training of HCWs to prevent MPXV transmission in healthcare settings, especially during specimen collection (*5*).
